# What parents tell us about the complex reality of lethal means restriction

**DOI:** 10.1192/bjo.2025.10899

**Published:** 2025-11-10

**Authors:** Rachel Kritzik, Bonnie Klimes-Dougan, Kathryn R. Cullen

**Affiliations:** Institute of Child Development, University of Minnesota, Minneapolis, MN, USA; Department of Psychology, University of Minnesota, Minneapolis, MN, USA; Masonic Institute for the Developing Brain, University of Minnesota, Minneapolis, MN, USA; Department of Psychiatry and Behavioral Sciences, Medical School, University of Minnesota, Minneapolis, MN, USA

**Keywords:** Suicide, qualitative research, prevention, self-harm, risk assessment

## Abstract

Through rich qualitative interviews, Simon and colleagues highlight how parents of suicidal adolescents navigate the process of lethal means restriction (LMR). Parents face challenges throughout the course of LMR that impact not only their ability to implement it effectively, but also the family dynamic at large. Results underscore a need for standardised, comprehensive training in LMR for clinical and medical professionals, as well as for policy solutions that can have more widespread influence and reduce the burden on parents as they support their children through an extraordinarily difficult time.

Suicide is one of the leading causes of death in adolescents and young adults worldwide, but it can also be highly preventable.^1^ Adolescence represents a particularly salient developmental period for studying suicide prevention, because rates of suicide attempts and fatality increase significantly from early and middle childhood to adolescence.^2^ Factors such as heightened impulsivity, increased independence, greater substance use and higher rates of mental health disorders may all contribute to these trends.^3^ During adolescence, suicide attempts become increasingly more lethal compared with those at younger ages, and this difference is particularly pronounced for certain methods including drowning, hanging and jumping.^2^ According to contemporary suicide theories, the transition from suicidal ideation to action rests primarily on suicide capability, which can be thought of in terms of either acquired (i.e. habituation to pain or death), dispositional (i.e. biological or genetic contributors) or practical (i.e. knowledge of and access means) capabilities.^4^ Within research on such ideation-to-action frameworks, practical capability receives the least amount of theoretical attention yet it remains one of the most commonly recommended avenues for intervention via lethal means restriction (LMR).^5,6^

An empirically supported suicide prevention method, LMR focuses on restricting access to lethal means for people at risk for an attempt. LMR can be conducted at either the individual (e.g. removing firearms from the home) or societal (e.g. banning highly toxic pesticides) level.^7^ While long-term therapeutic support is essential when addressing suicidality, ensuring safety during acute and heightened periods of psychological distress is often most important in preventing suicide mortality. Despite LMR’s importance in preventing youth suicide attempts, less is known about how youths’ parents, who are often charged with implementing LMR and navigating these challenging demands. It is crucial to understand the rich and nuanced experiences of parents tasked with LMR to identify barriers and facilitators to its implementation.

In the current issue of *BJPsychOpen*, Simon and colleagues report results from a qualitative study that aimed to understand the emotional and relational impacts of LMR on parents of youth experiencing suicidality, and to identify the challenges and needs that parents have while implementing LMR.^8^ Twelve parents of Israeli adolescents and young adults aged 12–21 years with a history of suicidal ideation and/or behaviour participated in semi-structured interviews about their experiences with LMR. Researchers employed an interpretive phenomenological analysis approach to highlight the subjective meaning-making of their participants.

The researchers identified themes across two broad categories: the timeline of parents’ experiences with LMR and its effects on the family. Parents discussed key challenges at different stages of the LMR process, such as initially misinterpreting the guidance or having difficulty enforcing it – for example, due to privacy concerns or logistical barriers. Many parents felt a fear of responsibility for their child’s life when tasked with LMR. As time went on, other challenges arose because parents felt that LMR was not always sufficient to prevent a suicide attempt, that engaging in LMR meant treating their child as if they were much younger and that it led to personal costs including poorer mental health for their child, and to financial challenges for themselves. Some parents also discussed ways that they transitioned from LMR to more supportive roles because LMR felt exhausting, impossible, unhelpful or even harmful. LMR also affected the family as a whole. Some siblings experienced frustration at the situation, while others helped with LMR support. Likewise, some parent–child relationships were harmed due to breaches of trust, while other parent–child relationships improved as a result of the LMR process.

The results of this study highlight the complicated nature of LMR engagement for parents of youth experiencing suicidality. Different challenges arrive at different stages of the LMR process, affecting parents’ well-being and family dynamics. The qualitative nature of this study allowed for rich data and nuanced insights into a frequently neglected population of an already understudied area. Such person-centred, detailed experiences can offer important contributions to the literature in ways that are hard to capture through quantitative methods. Important future steps would introduce quantitative methodologies to test hypotheses that were generated through this qualitative work.

As the authors note, access to firearms is heavily restricted in Israel; furthermore, adolescent suicide rates in Israel are among the lowest of peer countries.^9^ In the USA, for example, firearms are the leading cause of death in children, including suicide mortality.^10^ Firearms are also the most lethal method of suicide and remain relatively consistently lethal across childhood and adolescence.^2^ Therefore, firearms are one of the most important targets for LMR and, while not discussed in the current study, one can imagine that the challenges faced by parents tasked with LMR are compounded when firearms are in the picture. For example, parents often mistakenly believe that their children cannot access firearms in the home, highlighting an important opportunity for prevention, including LMR.^11^ Examination of parental experiences with LMR in preventing teen suicide in other contexts with higher access to firearms and higher rates of suicide will be important for future research.

The results reported by Simon and colleagues have important clinical and policy implications. From a clinical perspective, reported phenomena, such as parents misinterpreting the guidance or believing that LMR could actually be harmful, suggest a need for standardised, comprehensive training in LMR for clinical and medical professionals and ongoing family input. Common misconceptions and concerns about LMR should be routinely addressed during LMR guidance. Furthermore, the results of this study by Simon and colleagues demonstrate how the entire family is affected when tasked with LMR. Parents reported increased anxiety, fear and hopelessness, as well as frustrations from siblings. Therefore, clinical interventions aiming to support suicidal youth should take the family context into account, and additional therapeutic support should be routinely offered to help parents as they navigate the LMR process. To further reduce the burden on parents, LMR should also be approached from a policy perspective. This can be at the local level (e.g. installing a fence or net at a bridge), the state level (e.g. implementing Child Access Prevention laws, which make it illegal for an adult to let a child access a firearm) or the national level (e.g. banning highly toxic pesticides). Such policy initiatives show promise in preventing suicide, and can do so at a larger scale than individual LMR.^12^

In conclusion, LMR is an important intervention in youth suicide prevention but, as demonstrated by Simon and colleagues, parents face many nuanced challenges that must be addressed before it can be maximally effective. Consideration of the unique regional contexts will be critical for enhancing the efficacy of LMR in the future.
